# Outcomes in AB0 Incompatible Living Donor Kidney Transplantation: A Case – Control Study

**DOI:** 10.3389/fmed.2022.932171

**Published:** 2022-07-22

**Authors:** Martina Cozzi, Paola Donato, Gabriele Ugolini, Rostand Emmanuel Nguefouet Momo, Francesco Nacchia, Zeno Ballarini, Pierluigi Piccoli, Maurizio Cantini, Chiara Caletti, Stefano Andreola, Giorgio Gandini, Giovanni Gambaro, Luigino Boschiero

**Affiliations:** ^1^Kidney Transplant Center, Department of Surgical Sciences, University and Hospital Trust of Verona, Verona, Italy; ^2^Nephrology Postgraduate School, Department of Medicine, University of Verona, Verona, Italy; ^3^Transfusion Medicine Unit, Department of Pathology and Diagnostic Services, University and Hospital Trust of Verona, Verona, Italy; ^4^Renal Unit, Department of Medicine, University and Hospital Trust of Verona, Verona, Italy

**Keywords:** AB0 incompatible, living donor kidney transplantation, desensitization, isoagglutinin titer, kidney transplant

## Abstract

**Background:**

Patients waiting for a kidney transplant by far exceed available organs. AB0 incompatible living donor kidney transplantation (AB0i LDKT) represents an additional therapeutic strategy, but with higher risk for complications. We aimed at evaluating outcomes of AB0i LDKTs compared to compatible (AB0c) controls at our Institution.

**Methods:**

Retrospective matched case – control study (1:2) comparing AB0i vs. AB0c LDKTs from March 2012 to September 2021. Considered outcomes: graft function, acute rejection, sepsis, CMV infection, BK virus reactivation, death-censored graft survival, patient survival.

**Results:**

Seventeen AB0i LDKTs matched to 34 AB0c controls. We found excellent graft function, comparable in the two groups, at all considered intervals, with an eGFR (ml/min/1.73 m^2^) of 67 vs. 66 at 1 year (*p* = 0.41), 63 vs. 64 at 3 years (*p* = 0.53). AB0i recipients had a statistically significant higher incidence of acute rejection, acute antibody-mediated rejection and sepsis within 30 days (*p* = 0.016; *p* = 0.02; *p* = 0.001), 1 year (*p* = 0.012; *p* = 0.02; *p* = 0.0004) and 3 years (*p* = 0.004; *p* = 0.006; *p* = 0.012) after surgery. There was no difference in CMV infection, BK virus reactivation, death-censored graft survival between the two groups. Patient survival was inferior in AB0i group at 1 and 3 years (88.2 vs. 100%; log-rank *p* = 0.03) due to early death for opportunistic infections. AB0i LDKTs spent longer time on dialysis (*p* = 0.04) and 82.3 vs. 38.3% controls had blood group 0 (*p* = 0.003).

**Conclusions:**

AB0i LDKT is an effective therapeutic strategy with graft function and survival comparable to AB0c LDKTs, despite higher rates of acute rejection and sepsis. It is an additional opportunity for patients with less chances of being transplanted, as blood group 0 individuals.

## Introduction

End stage renal disease (ESRD) represents a constantly growing health-care burden worldwide. Kidney transplantation stands as the best therapeutic option for patients with ESRD, with lower morbidity and mortality rates compared to dialysis (1). However, the number of patients waiting for a kidney transplant by far exceeds the number of available organs (2). In this scenario, strategies to answer a growing demand for transplantation are of vital importance and include the expansion of living donor kidney transplantation (LDKT) programs. Overcoming blood group incompatibility, the so-called AB0 barrier, signed a significant turning point in available therapeutic opportunities for our patients (3), as up to one third of them have an available, but incompatible, living donor. Moreover, not all patients have the same chances to receive a kidney from a deceased donor, with sensitized individuals and blood group 0 patients spending longer time on waiting list (2). The introduction of standardized desensitization protocols to remove anti-A/B antibodies and avoid hyperacute rejection allowed AB0 incompatible (AB0i) transplantation to become a well-established procedure with functional outcomes comparable to AB0 compatible (AB0c) LDKTs (4, 5). This procedure, however, has increased risk for complications, including antibody-mediated rejection and infection. We conducted a matched case-control study to evaluate outcomes of AB0i LDKTs compared to AB0c LDKTs performed at our Institution.

## Materials and Methods

### Study Population

All AB0i LDKTs performed at the Kidney Transplant Center of the University and Hospital Trust of Verona from March 2012 to September 2021 were enrolled as cases and matched to two AB0c LDKT controls. Matching was based on donor and recipient age, parental affiliation (blood related or non-related couples, parental degree), number of HLA mismatches and time of transplantation. Exclusion criteria for matching eligibility were: combined-organ transplant, a positive cross-match, a follow up shorter than 6 months, unavailability of complete data.

### Isoagglutinin Titers and Desensitization Protocol in AB0i LDKT

IgM and IgG isoagglutinin titers were measured using a conventional tube test for agglutination (6). Titers were measured at baseline, the day after every session of apheresis, the day of surgery, every other day during the first 2 weeks after surgery, at 1, 3, 6, 12 months, once a year after transplantation, and in case of acute rejection (AR). At all time-points, the higher level, either IgM or IgG, was considered.

All AB0i LKDTs received Rituximab, standard non-selective plasmapheresis (PP), IVIg and standard immunosuppression before transplantation. Rituximab (Roche; single dose - 375 mg/m^2^) was administered 30 days before the procedure. Sessions of PP were started two to three weeks before transplantation, on alternate days, aiming at achieving a titer ≤8. For PP, a centrifuge-driven cell separator was used, with 1 plasma volume removed per each session and replaced with albumin 5%. To reduce the risk of bleeding, fresh frozen plasma was used in the 2 sessions before transplantation and in additional PP after surgery. From January 2017, routine PP after transplantation were omitted and performed “on demand” in case of titer rebound (≥16) or acute antibody-mediated rejection (aAMR). The day after PP, high dose IVIg (Privigen, CSL Behring; 2 g/kg) were administered until transplantation. Two weeks before transplantation immunosuppression therapy with tacrolimus (Astellas; target trough level 8–10 μg/L), mycophenolate sodium (Novartis; 720 mg twice daily), and methylprednisolone (16 mg per day) was started.

### Immunosuppressive Therapy and Prophylaxis

All AB0i and AB0c LDKTs received, as induction therapy, either Basiliximab (Novartis; 20 mg on day 0 and 4 post op) or rabbit anti-thymocyte globulin (rATG; Neovii; 2 mg/kg on day 0, 2, 4 post op), and methylprednisolone (10 mg/kg i.v. intraoperatively, tapered to 16 mg by day 7 post op). Tacrolimus, mycophenolate sodium and steroids were used as maintenance therapy. All patients received valganciclovir (Roche) for 3–5 months as prophylaxis for cytomegalovirus (CMV) infection, according to donor–recipient serological status, and trimethoprim/sulfamethoxazole or dapsone as prophylaxis for *Pneumocystiis jirovecii* (PCP) for 3 months after transplantation. No routine systemic fungal prophylaxis was used.

### Clinical Data, Primary and Secondary Outcomes

For each donor – recipient couple we registered: age, sex, blood group, number of HLA mismatches, parental relationship. For all donors: baseline serum creatinine (SCr), estimated glomerular filtration rate (eGFR), hypertension. For all recipients: cause of ESRD, presence of diabetes mellitus, type and duration of renal replacement therapy, panel-reactive antibody (PRA), donor-specific antibodies (DSA), anti-HLA non DSA antibodies, previous transplants.

We considered as primary outcomes graft function and the following complications: delayed graft function (DGF), acute rejection (AR), sepsis, viral infections (CMV, BK virus). For graft function, SCr and eGFR were considered at 1, 3, 6 months, 1, 3 years of follow-up. eGFR was calculated with CKD-EPI 2021 equation (7). All “complications” events were considered within 30 days, 1 and 3 years after transplantation. DGF was defined as the need of at least one dialysis session in the first week after transplantation. Diagnosis of AR was biopsy-proven and scored according to the Banff Classification (8) as acute antibody-mediated rejection (aAMR) or acute T cell-mediated rejection (aTCMR). Sepsis events were defined as bacterial/fungal infections with positive blood stream cultures and/or the need of hospital re-admission. Type of infection was recorded. Uncomplicated urinary tract infections were not considered as events. CMV infection was defined as detection of plasmatic CMV DNA copies associated with symptomatic infection and treated with antiviral therapy. BK virus reactivation was defined as plasma viral load >10^3^ copies/mL. As secondary outcomes, we considered death-censored graft survival and patient survival at 1 and 3 years of follow up.

### Statistical Analysis

Means and standard deviations were used for the description of continuous variables. Categorical variables were expressed in numbers and percentages and divided in classes. Student's *T*-test/one-way ANOVA was used to compare means. Chi-squared test and Fisher exact test were used to compare categorical variables. Survival functions were plotted on Kaplan–Meier curves and compared using log-rank test. A *p* value < 0.05 was considered for statistical significance. All data were processed using Stata 14.1 for Windows (StataCorp, College Station, Texas).

## Results

### Study Population

Between March 2012 and September 2021, 155 LDKTs were performed at our Institution. Seventeen were AB0i LDKTs, enrolled as cases and matched to 34 AB0c controls. There was no difference in donors' basal kidney function between the two groups. Two AB0i recipients had a history of previous transplant, two had a positive PRA, and one had positive DSA at the time of transplantation. One AB0c recipient had a positive PRA, none had DSA nor had a previous transplant. Eleven recipients (64.7%) in AB0i vs. 13 (38.2%) in the AB0c group were on hemodialysis, three (17.6%) vs. seven (20.6%) on peritoneal dialysis, three (17.6%) vs. 14 (41.2%) had a pre-emptive transplant. Mean time on dialysis before transplantation was 24.2 months in AB0i group vs. 9.9 months in AB0c group (*p* = 0.04). Blood type was 0 in 14 AB0i patients (82.3%) vs. 13 (38.2%) AB0c controls (*p* = 0.003). The following combinations of AB0i transplants were performed: 10 A to 0 (58.8%), 4 B to 0 (23.5%), 2AB to A (11.7%) and 1 AB to B (5.9%). Complete data on donors and recipients are summarized in Table 1.

**Table 1:** Baseline characteristics of recipients and donors in AB0i and AB0c groups.

	**AB0i** **(*n* = 17)**	**AB0c** **(*n* = 34)**	***p*-Value**
**Recipient characteristics**			
Male sex (%)	11 (64.7)	24 (70.6)	0.66
Age: mean (±SD), years	44.6 (10.7)	45.8 (13.4)	0.62
Diabetes mellitus, *n* (%)	0	1 (2.9)	1
**Cause of ESRD**, ***n*** **(%)**			
ADPKD	5 (29.4)	7 (20.6)	0.49
IgA nephropathy	3 (17.6)	11 (32.4)	0.26
ANCA vasculitis	3 (17.6)	0	0.03
HUS (typical)	0	1(2.9)	1
Vesicoureteral reflux	3 (17.6)	3 (8.8)	0.38
Inherited nephropathy	1 (5.9)	2 (5.9)	1
Interstitial nephritis	1 (5.9)	1 (2.9)	1
Other	0	4 (11.8)	0.28
Unknown	1 (5.9)	5 (14.7)	0.64
**Dialysis modality**, ***n*** **(%)**			
Hemodialysis	11 (64.7)	13 (38.2)	0.07
Peritoneal dialysis	3 (17.6)	7 (20.6)	0.77
None (pre-emptive)	3 (17.6)	14 (41.2)	0.09
Months on dialysis:	24.2 (±34.7)	9.9 (±14.8)	0.04
Previous transplant, *n* (%)	2 (11.8)	0	0.10
PRA >0%	2 (11.7)	1(2.9)	0.25
Anti – HLA antibodies (non DSA), *n* (%)	2 (11.7)	4 (11.7)	1
DSA, *n* (%)	1 (5.9)	0	0.34
HLA A + B + DR MM, mean (±SD)	3.7 (1.4)	3.6 (1.5)	0.39
**Blood group**, ***n*** **(%)**			
0	14 (82.3)	13 (38.3)	0.003
A	2 (11.7)	17 (50)	0.007
B	1 (5.9)	4 (11.7)	0.50
**Donor characteristics**			
Male sex (%)	6 (35.3)	10 (29.4)	0.67
Age: mean (±SD), years	52.8 (12.2)	52.2 (9)	0.43
Serum creatinine (μmol/L): mean (±SD)	73.9 (10.7)	69 (13.7)	0.10
eGFR: mean (±SD)	94 (13.1)	97.8 (13.1)	0.16
Hypertension, *n* (%)	2 (11.7)	5 (14.7)	0.77
Blood related couples, *n* (%)	7 (41.2)	14 (41.2)	1
Mother → Son/Daughter	5	10	
Blood unrelated couples, *n* (%)	10 (58.8)	20 (58.8)	1
Wife → Husband	5	13	
Husband → Wife	5	7	
**Induction therapy**, ***n***			
Basiliximab	16	33	0.7
rATG	1	1	0.7
Mycophenolate sodium	17	34	1
Methylprednisolone boluses	17	34	1

*ADPKD, autosomal dominant polycystic kidney disease; DSA, donor-specific antibodies; eGFR, estimated glomerular filtration rate; HLA, human leukocyte antigens; HUS, hemolytic uremic syndrome; PRA, panel-reactive antibody; rATG, rabbit anti-thymocyte globulins*.

### Isoagglutinin Titers in AB0i LDKT

**Figure 1:**
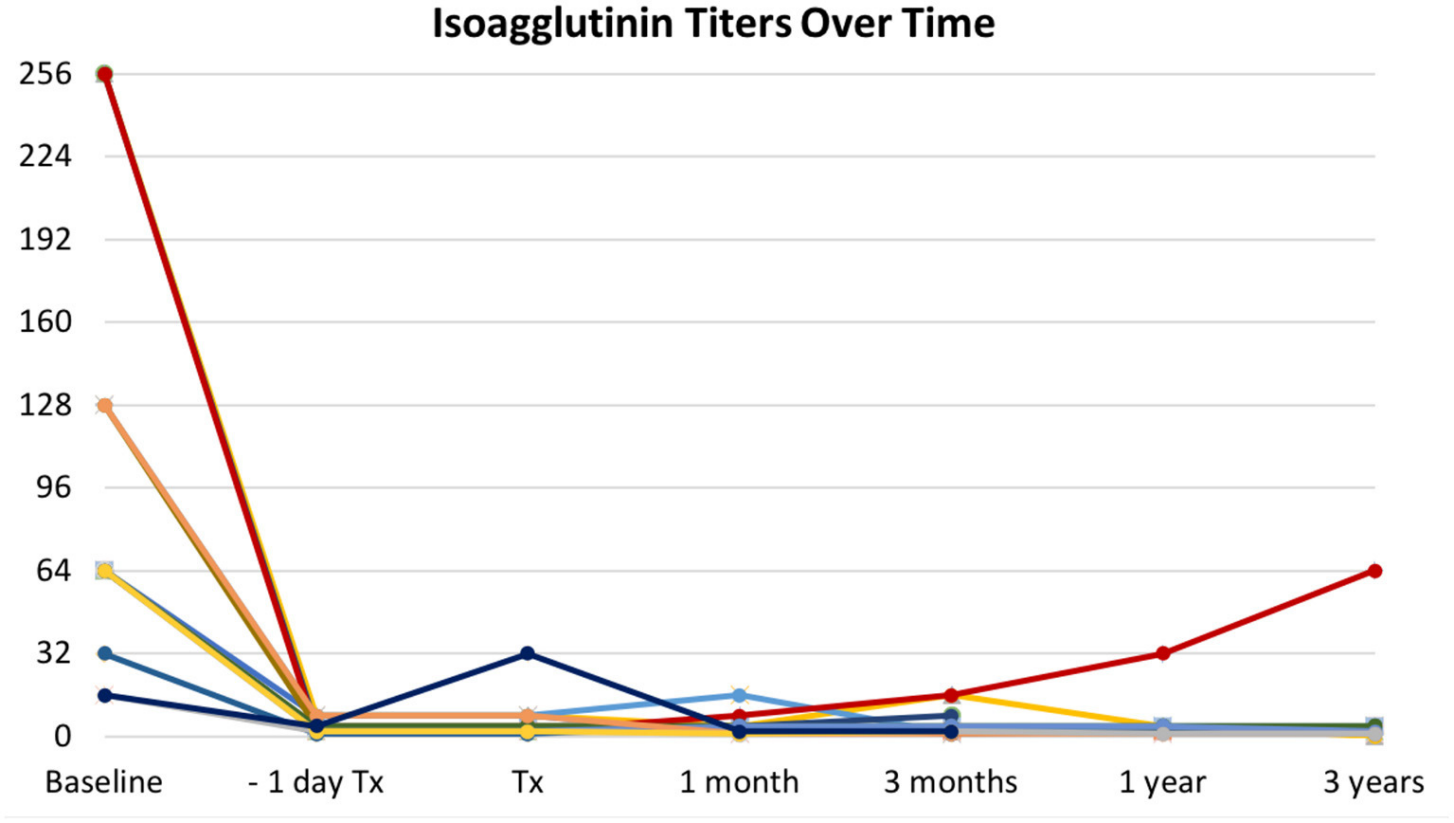
Trends in isoagglutinin titers over time. All patients successfully responded to desensitization protocol, with isoagglutinin titers abated within the day before transplantation (Tx). One patient experienced a sudden rebound the day of surgery (dark blue line). Titers remained stably low or slowly increased during follow-up, with no associated antibody-mediated rejection, accounting for what is defined as accommodation. Different colors in lines account for patients grouped according to the same baseline titers.

Basal isoagglutinin titer was **≥**64 in 14 patients (82.3%), being 256 in three patients (17.6%) and 128 in other three (17.6%). All patients achieved a titer ≤8 within the day before surgery (Figure 1). The mean number of PP sessions before transplantation was 7.5 (±2.9; range 3–13), with more sessions needed for higher baseline titers. Nine patients received additional PP after transplantation, four as scheduled procedure, four for aAMR, one for titer rebound from 4 to 32 on the day of surgery. Rebound was detected after scheduled administration of IVIg and did not lead to AR. During follow-up, titers remained low or slightly increased with no associated AR or worsening of graft function (Figure 1).

### Graft Function

**Figure 2:**
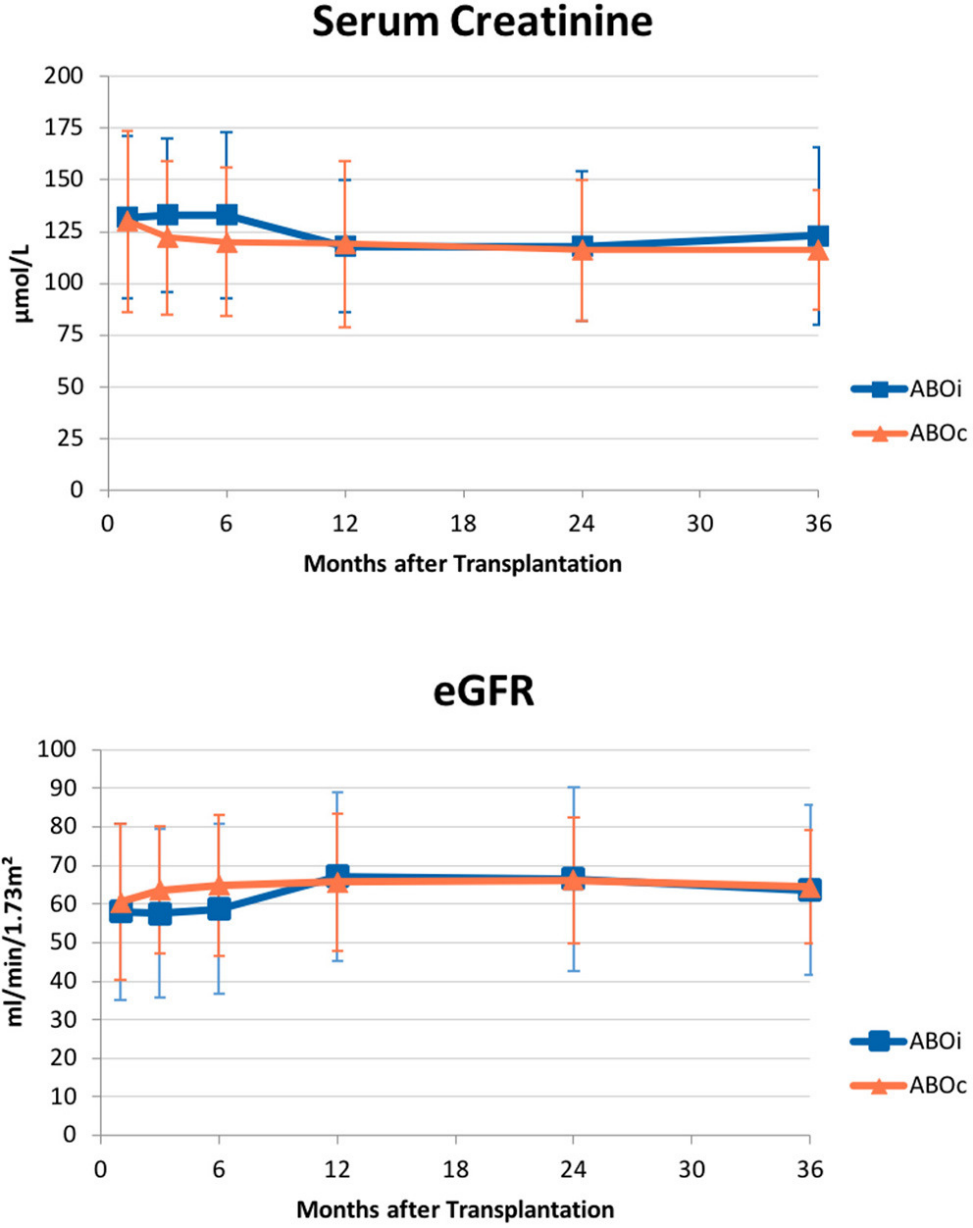
Graft function over time. Mean (±SD) serum creatinine (upper panel) and estimated glomerular filtration rate (eGFR; lower panel) did not differ in AB0 incompatible patients and compatible controls at all considered intervals – 1, 3, 6 months, 1, 2, 3 years from transplantation.

There was no statistically significant difference in graft function between the two groups at all considered time points, with excellent functional outcomes. Mean SCr (μmol/L) was 132 vs. 130 at 1 month (*p* = 0.49), 118 vs. 119 at 1 year (*p* = 0.53), 123 vs. 116 at 3 years (*p* = 0.47) in AB0i and AB0c LDKTs, respectively. Mean eGFR (ml/min/1.73 m^2^) was 57 vs. 60 at 1 month (*p* = 0.28), 67 vs. 66 at 1 year (*p* = 0.41), and 63 vs. 64 at 3 years (*p* = 0.53) in AB0i vs. AB0c LDKTs. Complete data are shown in Figure 2.

### Complications

We registered only one case of DGF, an AB0i LDKT with systemic de novo thrombotic microangiopathy (sdTMA) and aAMR. Patient's isoagglutinin titer was 128 at baseline and 8 at the time of surgery. Therapy with Eculizumab, PP, and methylprednisolone boluses was effective, with a regain of function and an eGRF of 34 ml/min 1.73 m^2^ at 1 month, stable at 3-year follow up.

AB0i recipients had a statistically significant higher rate of AR and aAMR at 30 days, 1 and 3 years after transplantation, compared to AB0c controls. Within 30 days, 7 (41.2%) vs 4 (11.8%) AR events occurred in AB0i vs AB0c LDKTs (*p* = 0.016), 4 (23.5%) vs 1 (2.9%) aAMR (*p* = 0.02). Within 1 year, 8 (47%) vs 5 (14.7%) AR events were registered in AB0i vs AB0c controls (*p* = 0.012), 5 (29.4%) vs 2 (5.9%) aAMR (*p* = 0.02). After 3 years, 9 (52.9%) vs 5 (14.7%) AR events occurred in AB0i vs AB0c controls (*p* = 0.004), 6 (35.2%) vs 2 (5.9%) aAMR (*p* = 0.006). All AR events in both groups were responsive to therapy and did not lead to graft loss. None of the AR events in the AB0i group was associated with a rebound in isoagglutinin titer.

Sepsis events, as well, were significantly more frequent in AB0i cases vs. AB0c controls at all considered time-points. Six (35.3%) AB0i vs. 1 (2.9%) AB0c LDKTs developed a sepsis (*p* = 0.001) within 30 days after surgery, 8 (47%) vs. 2 (5.9%) at 1 year (*p* = 0.0004), and 8 (47%) vs. 5 (14.7%) at 3 years (*p* = 0.012). Urosepsis was the most frequent recorded infection in both groups. Six of the 8 AB0i LDKTs who had a sepsis, had an AR and had received additional immunosuppressive therapy before sepsis onset.

There was no statistically significant difference in the incidence of CMV infection and BK virus reactivation between the two groups. Complete data on complications are summarized in Table 2.

**Table 2:** Complications registered within 1 month, 1 year and 3 years after transplantation in AB0i and AB0c recipients.

**Complications**	**AB0i** **(*n* = 17)**	**AB0c** **(*n* = 34)**	***p*-Value**
DGF	1	0	0.34
Acute rejection, *n* (%)			
Within 30 days	7 (41.2)	4 (11.8)	0.016
aAMR	4 (23.5)	1 (2.9)	0.02
aTCMR	3 (17.6)	3 (8.8)	0.35
Within 1 year	8 (47)	5 (14.7)	0.012
aAMR	5 (29.4)	2 (5.9)	0.02
aTCMR	3 (17.6)	3 (8.8)	0.35
Within 3 years	9 (52.9)	5 (14.7)	0.004
aAMR	6 (35.2)	2 (5.9)	0.006
aTCMR	3 (17.6)	3 (8.8)	0.35
Pts with a sepsis event, *n* (%)			
Within 30 days	6 (35.3)	1 (2.9)	0.001
Within 1 year	8 (47)	2 (5.9)	0.0004
Within 3 years	8 (47)	5 (14.7)	0.012
Pts with >1 sepsis, *n*	4 (23.5)	0	0.009
Tot. Sepsis events, *n*	15	5	0.003
Urosepsis, *n* (%)	13 (86.7)	4 (80)	0.71
PCP, *n* (%)	1 (6.7)	0	1
Other, *n* (%)	1 (6.7)	1 (20)	0.44
Deaths for infection, *n*	2	0	0.10
BK virus replication, *n* (%)	2 (11.7)	4 (11.7)	1
CMV infection, *n* (%)	3 (17.6)	2 (5.9)	0.18
Primary infection, *n*	2	1	
Reactivation, *n*	1	1	

*aAMR, acute antibody mediated rejection; aTCMR, acute T-cell mediated rejection; DGF, delayed graft function; PCP, Pneumocystis jirovecii Pneumonia; CMV, cytomegalovirus*.

### Death-Censored Graft Survival and Patient Survival

No patient experienced graft loss due to rejection, infection or other causes, with an identical death-censored graft survival of 100% in the two groups at 3 years. Patient survival was inferior in AB0i group at 1 and 3 years (88.2% vs. 100%; log-rank *p* = 0.03), as two AB0i recipient died in the first 6 months after transplantation due to infection. The first patient died after 47 days due to *P. jirovecii* pneumonia, while the second patient died after 138 days for disseminated fungal infection.

## Discussion

We conducted a matched case-control study to evaluate outcomes of AB0i vs. AB0c LDKTs performed at our Institution. First of all, we found excellent graft function in AB0i recipients at all considered intervals, from 1 month to 3 years after transplantation, comparable to AB0c controls. This finding is in line with what reported by other studies, including experiences from Heidelberg (9, 10), Freiburg (11) and the UK (12).

Only one patient, an AB0i recipient, experienced a DGF with an underlying sdTMA effectively treated with Eculizumab and additional PP. It has been demonstrated that AB0i transplantation constitutes an independent risk factor for sdTMA and that an isoagglutinin titer ≥64 before desensitization therapy, as it was in our patient, is a significant risk factor, despite successful desensitization before surgery (13). Efficacy of Eculizumab and apheresis for sdTMA in AB0i transplantation has been documented also by other case-reports (14).

In our study, AB0i recipients had higher incidence of AR and aAMR compared to AB0c controls, with the majority of events registered within 30 days after surgery. Patients undergoing AB0i transplantation have a higher immunological risk due to the procedure itself. The risk further increases in individuals with a history of sensitization, which is not infrequent in patients undergoing AB0i LDKT. Nonetheless, literature data on AR in these patients are conflicting. The two largest meta-analysis on AB0i LDKTs performed to date, including 1,346 and 7,098 cases, respectively, both reported higher risk of AR and aAMR in AB0i recipients compared to compatible controls (15, 16). In a Johns Hopkins study on the risk of aAMR in AB0i transplantation (17), 26% of the 115 enrolled patients experienced aAMR, 49% within 30 days after transplantation. On the other hand, other single-center studies did not observe differences in the incidence of AR and aAMR between AB0i and AB0c LDKTs (9, 11, 12). These conflicting results can be explained considering different definitions of AR among studies, not all being biopsy-proven, and different cohorts, not comparable for baseline characteristics and used immunosuppressive therapy.

In our study, as in the above-mentioned studies, AR events in AB0i LDKTs were not linked to a rebound in isoagglutinin titer. The only rebound that we observed was detected the day after administration of scheduled IVIg. It is known that IVIg may contain anti-A/B isoagglutinins, and this could justify the sudden and unexpected rebound after an effective desensitization (18). The rebound was treated with additional PP and did not elicit an AR. During the 3-year follow up, in all AB0i recipients titers remained low or slightly increased without worsening in graft function, accounting for what is known as accommodation (19).

Another main focus of our investigation were infections. AB0i recipients had a significantly higher rate of sepsis at all considered time points, with two fatal events from opportunistic infections in the first 6 months after transplantation. These findings are in line with literature data. Both the above mentioned meta-analysis showed increased incidence of sepsis in AB0i LDKTs (15, 16). Another study on 119 AB0i recipients from U.S. Renal Data system showed a statistically significant higher incidence of infections within 90 days from transplantation, compared to AB0c controls (20), as it was in our study. In AB0i LDKTs, increased risk of sepsis can be related not only to desensitization therapy, but also to a stronger immunosuppression regimen maintained after transplantation, and to additional immunosuppressive therapies administered in case of AR, which is not an infrequent event, as we discussed.

With regards to viral infections, we observed no differences in CMV infection and BK virus reactivation between the two groups. However, the small number of observed events does not allow to draw solid conclusions and divergent data on this topic are reported also by the current literature. Scurt et al. (16) meta-analysis showed no difference in the risk of CMV and BK infections between AB0i and AB0c controls, while De Weerd et al. (15) meta-analysis reported slightly more frequent CMV viremia in AB0i recipients, and conflicting results on BK virus, with studies reporting either higher or lower incidence of BK viremia and BK associated nephropathy. These divergent results probably originate from differences in studied cohorts and definitions of the events.

Considering secondary outcomes, we found excellent death-censored graft survival, with no difference between the two groups, in line with the literature. The European Collaborative Transplant Study (CTS) (21), one of the largest registry to date, analyzing outcomes of 1,420 AB0i recipients and matched AB0c controls, reported identical death-censored graft survival in the two groups at 3 years, as in our study. Other studies from different Countries confirmed the same results (12, 22, 23). The U.S. Scientific Registry of Transplant Recipients, with 738 AB0i LDKTs and matched controls, recorded a statistically significant lower graft survival in AB0i recipients, mainly driven by graft loss in the first two weeks after transplantation, while, after 14 days from surgery, graft survival became similar to that of AB0c controls on a long-term follow up (24). Scurt et al. (16) meta-analysis reported an inferior death-censored graft survival at 1 and 3 years. However, subgroup analysis demonstrated there was no difference in death-censored graft survival in patients preconditioned with Rituximab, as in our patients, while graft survival was inferior in those not treated with Rituximab (16).

In our investigation, AB0i LDKTs had an inferior patient survival due to early death for opportunistic infections within the first year after transplantation, a finding reported also by other studies in the literature and to be considered as directly related to higher immunosuppression implied in AB0i transplantation. In De Weerd et al. meta-analysis, AB0i recipients had a lower 1-year patient survival (96% vs. 98%), with 49% of deaths caused by infectious events compared to only 13% in AB0c controls (15). The CTS study showed one additional death of infective origin per 100 AB0i recipients during the first year after transplantation. However, at 3 years there was no difference in survival between AB0i and AB0c LDKTs (21). Comparable long-term survival between AB0i and AB0c LDKTs was reported by the U.S. Scientific Registry (24) and by other single-center studies, as well (11, 22). Available evidences, although not conclusive, seem to show a worse outcome during the first year after transplantation, while on the long-term patient survival becomes comparable in AB0i and AB0c recipients. Of note, it has been recently demonstrated that, despite the entailed risks, also AB0i LDKT confers to recipients a survival benefit, with a lower 25% mortality risk, compared to staying on dialysis (25).

Finally, in our study, AB0i recipients had mostly blood group 0 (82.3%), spent longer time on dialysis before transplantation, had a lower rate of pre-emptive transplantation, a history of sensitization. All these data underline how AB0i transplantation represents an additional therapeutic option for patients with reduced chances of receiving an organ (26).

Considering risks of AB0i LDKT, an alternative option for AB0i pairs to be mentioned is kidney paired donation (KPD), a well-established strategy to overcome AB0 and HLA incompatibility barriers without desensitization (27). In KPD, two or more incompatible donor-recipient couples are matched in order to perform compatible LDKTs. It has been demonstrated that graft and patient survival outcomes are comparable in KPD and control LDKTs (28). However, while avoiding desensitization and the risks of incompatible LDKT, KPD may present organizational issues that could extend the waiting time before transplantation, with other consequent risks. In fact, this may lead to changes in recipients' clinical status, as they stay on dialysis while waiting, or changes in donors' status or willingness to donate, with recipients left without an organ (29). Most of all, KPD might be not a suitable solution for blood group 0 recipients of AB0i pairs, who need to find a matching couple with a blood group 0 donor. This donor is of course AB0c with his/her direct recipient, but HLA incompatible (HLAi). As recipients of HLAi pairs are often sensitized individuals, finding a suitable matching might be difficult, and more than two couples may need to be involved, with consequent organizational problems and entailed risks (29). A recent report on outcomes of 1,121 patients enrolled in a U.S. based KPD program found that waiting time for blood group 0 recipients was five-time longer compared to blood group A recipients (335 vs. 73 days), and even longer for sensitized blood group 0 individuals (671 vs. 420 days) (30). As we discussed, more than 80% of the recipients in our study had blood group 0. All in all, choosing between desensitization and alternative strategies should take into consideration patients' characteristics, risk for infection, immunological risk, morbidity and mortality risks related to staying on dialysis while waiting, and organizational issues, and it is not an easy decision to be made.

Our study has some limitations, being a single-center, retrospective, observational study with a limited number of cases and a medium-term follow up. However, as point of strength, the matching criteria, the analogous immunosuppressive induction therapy and prophylaxis for infection made the comparison between the two groups more robust.

In conclusion, we observed excellent outcomes in terms of graft function and graft survival in AB0i LDKTs on a medium term follow up, comparable to AB0c controls. During the first month and first year after transplantation, AB0i recipients experienced a higher burden of AR and sepsis, which, however, did not lead to graft loss. AB0i transplantation represents a viable therapeutic option for individuals with an available, but incompatible living donor, and for patients with reduced chances of being transplanted, including blood group 0 individuals. Further investigation and development in this field is warranted, as AB0i LDKTs represent one of the possible strategies to answer a constantly growing demand for transplantation.

## Data Availability Statement

The raw data supporting the conclusions of this article will be made available by the authors at request, without reservation.

## Ethics Statement

The study was conducted ethically in line with the principles of the Declaration of Helsinki.

## Author Contributions

MC, LB, and PP: research idea and study design. MC, PD, GU, RN, FN, ZB, and PP: data acquisition. MC, LB, PP, and GGam: data analysis/interpretation. LB: statistical analysis. MC and LB: wrote the manuscript. All authors reviewed the article and approved the final version.

## Conflict of Interest

The authors declare that the research was conducted in the absence of any commercial or financial relationships that could be construed as a potential conflict of interest.

## Publisher's Note

All claims expressed in this article are solely those of the authors and do not necessarily represent those of their affiliated organizations, or those of the publisher, the editors and the reviewers. Any product that may be evaluated in this article, or claim that may be made by its manufacturer, is not guaranteed or endorsed by the publisher.

## Abbreviations

AB0i: AB0 incompatible
AB0c: AB0 compatible
aAMR: acute antibody mediated rejection
AR: Acute rejection
aTCMR: acute T-cell mediated rejection
BKAN: BK virus associated nephropathy
CMV: cytomegalovirus
DSA: donor specific antibodies
eGFR: estimated glomerular filtration rate
ESRD: end stage renal disease
KPD: kidney paired donation
HLA: human leukocyte antigen
LDKT: living donor kidney transplantation
PP: plasmapheresis
PRA: panel-reactive antibody
PCP: *Pneumocystis jirovecii* Pneumonia
SCr: serum creatinine.
